# Modeling of post-traumatic epilepsy and experimental research aimed at its prevention

**DOI:** 10.1590/1414-431X202010656

**Published:** 2020-12-18

**Authors:** A.C. Mosini, M.L. Calió, M.L. Foresti, R.P.S. Valeriano, E. Garzon, L.E. Mello

**Affiliations:** 1 Departamento de Fisiologia, Universidade Federal de São Paulo, São Paulo, SP, Brasil; 2 Associação Brasileira de Epilepsia, São Paulo, SP, Brasil; 3 Instituto D'Or de Pesquisa e Ensino, Rio de Janeiro, RJ, Brasil; 4 Divisão de Clínica Neurológica, Faculdade de Medicina, Universidade de São Paulo, São Paulo, SP, Brasil

**Keywords:** Epileptogenesis, Animal models of epilepsy, Traumatic brain injury, Status epilepticus, Kindling, Seizure

## Abstract

Research on the prevention of post-traumatic epilepsy (PTE) has seen remarkable advances regarding its physiopathology in recent years. From the search for biomarkers that might be used to indicate individual susceptibility to the development of new animal models and the investigation of new drugs, a great deal of knowledge has been amassed. Various groups have concentrated efforts in generating new animal models of traumatic brain injury (TBI) in an attempt to provide the means to further produce knowledge on the subject. Here we forward the hypothesis that restricting the search of biomarkers and of new drugs to prevent PTE by using only a limited set of TBI models might hamper the understanding of this relevant and yet not preventable medical condition.

## Introduction

Traumatic brain injury (TBI) is a global significant cause of death and lifelong disability resulting in cognitive, physical, behavioral, and subjective sequela. It has been estimated that TBI affects over 10 million people annually worldwide, leading to either high mortality or hospitalization rates (1). Age-standardized TBI prevalence has grown 8.4% from 1990 to 2016 (2), and it is still considered a silent epidemic (3). Among younger adults, especially men, TBI is a major source of years lived with disability. In addition, reflecting the ageing global population, even more so in high-income countries, in recent decades there has also been an increase in the number of TBI occurrence in elderly people, mainly related to falls (2).

TBI is defined as an alteration in brain function, or other evidence of brain pathology, caused by an external mechanical force, such as an impact, rapid acceleration or deceleration, blast waves, crush, or penetration by an object, resulting in focal or diffuse types of injury (4). As presented, TBI is a heterogeneous condition, which embraces different causes, severity levels, and consequently a wide range of prognosis. Patients with moderate to severe TBI have a high overall mortality, and more than 40% of survivors experience long-term disabilities (5). Even mild TBI has been associated with several long-term adverse outcomes. Disabilities can manifest as physical and cognitive deficits (for example, transient confusion, disorientation, and impaired attention), psychological health issues (such as depression), and impairments in self-regulatory behaviors (such as increased impulsivity, poor decision-making, and aggressive behavior) (3). TBI is also the main cause of acquired epilepsy (6).

Epilepsy is defined as a disorder of the brain characterized by an enduring predisposition to generate epileptic seizures, and by the neurobiological, cognitive, psychological, and social consequences of this condition (7). Post-traumatic epilepsy (PTE) accounts for 5% of all epilepsy etiologies, making TBI one of the most important causes of secondary epilepsy, overcoming other causes such as infections and central nervous system tumors (8).

In spite of the high PTE incidence after TBI, the latency from injury to development of epilepsy is variable and can range from weeks to years (9). Seizures occurring in the first 1-2 weeks after trauma may be due to the acute effects of the trauma, such as hemorrhage and brain edema, and do not necessarily characterize epilepsy. Even so, the presence of acute seizures occurring within weeks after TBI is often thought to be associated with the progression to PTE further along in life. The recurrence of epileptic seizures within the first 2 years in TBI patients with a single acute seizure after trauma might be as high as 86% (10). The causative links between TBI and epilepsy, as well as other types of epilepsy in general, are still not completely understood, and PTE is not yet preventable.

Epileptogenesis is a dynamic process occurring between the initial brain insult and the onset of epilepsy, characterized by the development of the first spontaneous seizure. The epileptogenic process can be divided into three different stages: 1) Initial insult: primary injury by different insults, including the already mentioned TBI, and also status epilepticus (SE), febrile seizures, stroke, craniotomy, infections, tumors, and neurodegenerative diseases, among others; 2) Latent period: the primary injury triggers a cascade of molecular, cellular, and structural changes that include increased neuronal excitability and inflammatory processes, which contribute to additional neurodegeneration, and reorganization of molecular and cellular pathways, including gliosis, plasticity, neurogenesis, and mossy fiber sprouting (11,12). All these processes are supposed to be related, with greater or lower importance, to the development of the epileptic focus; and 3) Chronic epilepsy phase: clinical manifestation of spontaneous recurrent seizures.

Therefore, different insult types can be used to mimic specific characteristics of the primary lesion, and the subsequent epileptogenic mechanisms can be further studied during the latent period in different models of PTE.

The goal of this review is to describe the main animal models currently used for the study of PTE and to critically compare those models with regard to the development of the most common features present in this pathology. We propose that the onset of spontaneous and recurrent seizures, regardless of the initial stimulus, should be the most relevant feature of animal models used for the study of PTE. We suggest that the epileptogenesis associated with different etiologies are often very similar and that the investigation of potential means of disease modification or prophylaxis, in the case of PTE, can rely not only on TBI models, but also on a broader set of epilepsy models.

## Animal models of post-traumatic epilepsy

It is a great achievement for science to reproduce human diseases in animal models. A good animal model is homologous, able to mimic the human disorder and the physiopathological mechanisms in every respect. Alternatively, the animal model can be isomorphic, when it duplicates the disorder but not the underlying etiology, or predictive, in the case of models that cannot reproduce the human disorder but allow predictions about it or its response to treatment (13). As posed by the great mathematician Norbert Wiener "the best material model of a cat is another, or preferably the same, cat" (https://en.wikiquote.org/wiki/Norbert_Wiener). This review's whole argument is that, depending on the focus, the best model should mimic mechanisms and not form. To that end, here we evaluate both models of epileptogenesis, consisting of either repetitive stimulation of specific brain structures (kindling models) or the induction of intense prolonged acute seizures (SE models), as well as models developed for the study of PTE, using mechanical injuries (TBI models) or iron deposition (mimicking intraparenchymal bleeding). Other animal models for studying the epileptogenesis process, such as febrile seizures, models with transgenic animals, and stroke will not be explored in the current review. Indeed, this would not only substantially alter the scope of the current revision but would also lead to loss of the main argument we want to raise. Most groups using febrile seizures to study epilepsy are interested in developmental aspects of this condition and preferentially deploy juvenile animals. Similar limitations arise from the use of transgenic animals, which are usually structured as a means to investigate very specific contributions of a given protein or systems.

### TBI models

Animal models more directly related to TBI mechanisms were developed to investigate how the natural progression of trauma contributes to epileptogenesis. In a pioneer work, Willmore et al. were able to induce spontaneous seizures in animals over a period of a few weeks, by using a single iron injection to different brain regions (14). This injury model importantly relates to intracerebral hemorrhage toxicity caused by iron-rich hemoglobin breakdown products, similarly to what happens after a brain trauma or hemorrhagic stroke. It is known that FeCl_2_ does induce free radical formation, lipid peroxidation, and edema that can be attenuated by antiperoxidants. FeCl_2_ also causes alterations in glutamate transport (15). Recurrent seizures and epileptic discharge similar to human PTE have been seen after the generation of free radicals with the intracortical administration of ferric chloride into the sensorimotor cortex (16).

Thereafter, additional models mimicking different types of mechanical trauma have gained increased attention among experts investigating epileptogenesis associated with TBI. Among these, the fluid percussion injury (FPI), controlled cortical impact (CCI), weight-drop impact acceleration injury, and blast injury can be highlighted (4). Only a few models consider the importance of penetrating injury to epileptogenesis, such as the penetrating ballistic-like brain injury (17).

The lateral FPI model is the most utilized mechanical model to study PTE. By using a closed hydraulic system, a precise pressure pulse is delivered distinctly in the animal dura mater, according to the intended injury severity (18). In this model, a single event of lateral FPI is sufficient to induce chronic spontaneous recurrent partial seizures that worsen over time (19). According to D'Ambrosio and Perucca, lateral FPI can develop two epileptic foci depending on its location, one in the neocortex adjacent to the impact site that defines the early epileptic manifestations after injury, and one in the hippocampal region, which becomes increasingly dominant over time.

It is an easy to induce and highly reproducible model, but it has limitations such as high mortality in the severe injury conditions necessary to generate spontaneous seizures, increasing the total number of animals required for the PTE study. This model also promotes a low seizure frequency, and thus requires prolonged time of electroencephalogram (EEG) monitoring of spontaneous seizure events (20).

Another model used to directly mimic TBI is the CCI injury that was described by Lighthall (21) using ferrets as the test animal. During the 1990s, the model was adapted to be used in other animals like rats, mice, and primates. This model consists of a brain injury produced by a pneumatic or electromagnetic impact that compresses the exposed brain and results in brain injury with varying severity (18). Similar to FPI this model was created as a model of TBI and adapted for studying PTE.

As in the case of FPI, scarce late spontaneous seizures or epilepsy have been reported in rat CCI models (18,22). Despite the low frequency of spontaneous seizures, the model also leads to an increase in seizure susceptibility when challenged with other agents (23).

The CCI model provides consistency, reproducibility, with a low mortality rate compared to lateral FPI, being beneficial in reducing sample attrition and keeping study cost down. In contrast, this model includes mechanical variation, wear on the device, limited diffuse effects, and most importantly, it results in a low frequency of spontaneous chronic seizures, making it less useful for the study of epileptogenic mechanisms.

Impact-acceleration, also known as the weight drop model, is also a model of diffuse TBI. The animal is positioned on a foam block to provide a consistent position of the animal's body and a pre-selected weight is dropped from a pre-selected height according to the desired injury severity. The weight strike promotes not just a focal injury, but also promotes an acceleration-deceleration of the animal's head into the foam block (4).

The impact-acceleration model produces mild-to-moderate convulsions during its acute phase and may also cause late convulsions lasting up to 15 weeks post-injury. Although the model demonstrated an increase in susceptibility to pentylenetetrazol (PTZ)-evoked seizures, it fails to adequately recapitulate PTE because, without using a high intensity impact that frequently promotes great mortality, it does not promote spontaneous seizures (18). On the other hand, this model presents the advantages of simplicity of the method, successfully inducing a reproducible lesion and consistently increasing susceptibility to PTZ-induced seizures.

Another important model of TBI is the blast model that mimics a real blast-induced mild TBI, such as seen in military conflicts, and can be a potential risk factor for behavioral disorders, cognitive alterations, and for the development of neurodegenerative disorders. According to Song et al. (24), animals are allocated in open or closed shock tubes, or in an open field, 30 m away from a TNT explosive. Neocortical, hippocampal, and cerebellar tissue injuries have been reported in different degrees in the blast model together with vascular injuries, hemorrhage, and diffuse axonal injury (24). In the blast model, the occurrence of EEG epileptiform or PTE have not been generally assessed. Only a few studies show the occurrence of acute seizures following severe blast and there are no published studies investigating the occurrence of chronic (spontaneous) seizures or epileptogenic alterations in this model (25). The main advantage of the blast model is that it closely replicates the scenario of a real-world blast. On the other hand, this model is not ideal to investigate PTE, as it is expensive, it is difficult to find a safe place to store explosives, and the technical time demand and the environmental conditions such as temperature and wind may influence the animal responses and the blast setting (24).

Similarly, in the penetrating ballistic-like injury model, electrographic waveforms of ictal and inter-ictal events are reported, but occur later after injury and less frequently compared to those occurring in other injury models (26). In the repetitive weight-drop model and in the repetitive blast model, only a subset of mice (44 and 46%, respectively) develop spontaneous recurrent seizures (27).

**Figure 1:**
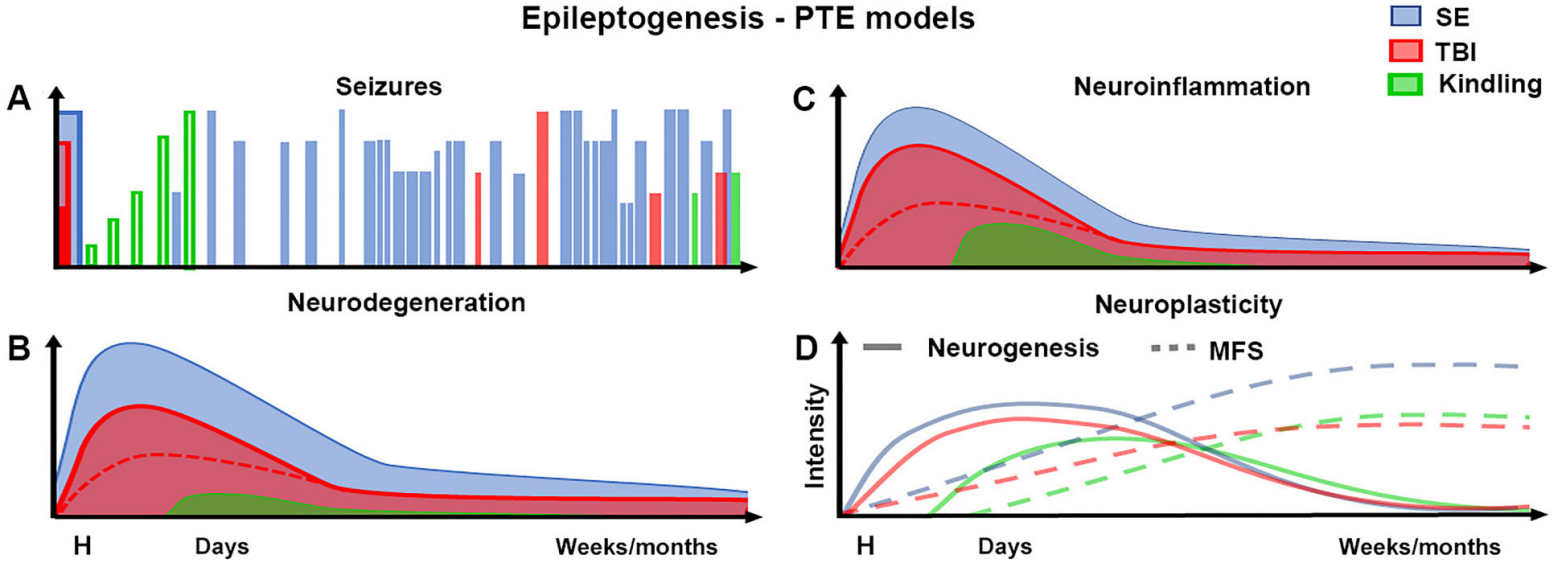
Schematic representation of epileptogenic features in main animal models of post-traumatic epilepsy (PTE). **A**, In classic models of status epilepticus (SE) (e.g., pilocarpine and kainic acid), after the initial sustained acute seizure, the development of spontaneous recurrent seizures occurs after a brief latent period in most animals. In contrast, the slow progression of evoked seizures in kindling models implies the need of massive repetition of the stimuli protocol in order for spontaneous seizures to develop, making this an extremely laborious protocol for that intention. Meanwhile, different parameters can be adjusted according to the desired lesion severity, and consequent occurrence or not of acute seizures, in different traumatic brain injury (TBI) models (e.g., lateral fluid percussion, controlled cortical impact, impact-acceleration, or weight drop). However, in TBI models, usually only a few animals, most of them requiring severe initial lesions, develop spontaneous recurrent seizures following a longer latent period. **B**, The lesion event may differently affect neurodegeneration occurrence. In general, chemically-induced SE causes severe and widespread neurodegeneration, while minor neuronal damage is detected in the kindling model. Importantly, by adjusting the mechanical force parameters, the neuronal damage can vary from cortical to subcortical regions, and from minor to severe neurodegeneration in TBI models. **C**, The neuroinflammatory response is rapidly initiated following the initial precipitating injury, characterized by the release of inflammatory cytokines, chemokines, and complement proteins. Astrocytes and microglial cells also became activated, proliferate, and, together with peripheral immune cells, are recruited to the lesion site. This response decreases over days, but residual neuroinflammation may chronically persist, supporting a pro-epileptogenic role. In general, the intensity of the neuroinflammation response can be considered quite similar between SE and TBI models, varying with the impact intensity in the latter. **D**, There is a transitory increase in newborn neurons in the subgranular zone of the dentate gyrus of the hippocampus and in the subventricular zone of lateral ventricles, in several animal models of PTE. After this initial increase, the number of newly generated cells returns to basal levels with the occurrence of spontaneous seizures. On the other hand, mossy fiber sprouting (MFS) increases after the initial insult and presents a permanent pattern together with spontaneous seizure occurrence.

As aforementioned, spontaneous epileptic activity, when present after mechanical injury, tend to show milder characteristics in terms of the epileptic events in TBI models (Figure 1). For example, the epileptogenesis phase in the FPI injury is longer, the resulting frequency of spontaneous seizures is substantially lower and occurs in fewer animals, and most of the seizures are secondarily generalized rather than partial, compared to epilepsy models that induce SE as the precipitating injury (28).

### Kindling model

One of the first and still widely used animal models to study epileptogenesis is the kindling model, which is characterized by the progressive development of focal seizures to secondarily generalized tonic seizures, after repeated application of an initially subconvulsive stimulus (29), which can be either electrical or chemical.

Electrical kindling applies subthreshold repetitive electrical stimuli in specific brain regions such as the dorsal hippocampus, olfactory bulb, amygdala, perirhinal cortex, and perforant path (30). Normally, these subthreshold stimuli, when administered occasionally, do not result in behavioral convulsive episodes, but, when administered repetitively, can promote behavioral convulsions. Rather than using electrical stimuli, chemical kindling involves repetitive administration of chemical convulsants. Convulsant substances such as cocaine, N-methyl-D-aspartate, and GABAa receptor antagonists, such as PTZ, can be used to induce chemical kindling. PTZ, one of the most widely used agents for the induction of chemical kindling, produces severe self-limited convulsions in animals.

In the kindling model, the seizure focus is explicit, seizures evolve gradually, and the development of chronic epileptogenesis can be investigated while not resulting in severe morphological damage (31). The progressive changes have a permanent feature, and although laborious, repeated stimulation for extended time can result in spontaneous seizures (32,33).

Unfortunately, this method demands resources and time, comprising long periods of handling and stimulation procedures (34). Still, many parameters of the kindling protocol have been modified to increase kindling representativeness as an epileptogenic model and to increase efficiency to assess antiepileptic drugs (AEDs). Such changes include but are not limited to: i) decreasing the interstimulus interval to shorten seizure development from weeks to hours (35); ii) selective breeding for segregating genetic predisposition to differential kindling rates (36); and iii) more recently, the application of the novel optogenetic approach to stimulate animals (37).

### SE models

The induction of SE, which is characterized by acute self-sustained seizures, is another classical experimental approach for the study of epileptogenesis. As in the kindling model, several stimuli can be used to trigger SE, including deep electrical stimulation to specific brain areas and systemic or topical injection of chemical agents, most notably kainic acid (KA) and pilocarpine (38–40). The early phase after the initial precipitating injury is associated with intense neuroplastic changes, neurodegeneration, and neuroinflammation. After a period past the SE event, spontaneous recurrent seizures easily develop together with behavior impairments associated with chronic inflammation, altered neurogenesis, abnormal synaptic reorganization, and multiple molecular changes.

The pilocarpine model consists in the systemic or topical (intrahippocampal) administration of this cholinergic agent, a potent muscarinic agonist. It promotes behavioral and electrographic alterations resulting in three different periods, which resemble the stages of the epileptogenesis process already described: i) Acute period characterized by SE originating in the limbic system and lasting up to 24 h, or until interruption with some specific drug; ii) Latent period, when the progressive normalization of behavior and EEG aspects and epileptogenic changes take place (this period has been reported to vary from 4-44 days); and iii) Chronic period, with the emergence of spontaneous recurrent seizures. The spontaneous seizures observed during the chronic period are similar to the complex partial seizures seen in patients. Seizure frequency in the chronic period is variable, some animals present a low seizure frequency over several weeks or months, while others may have daily seizures, and others may present seizures in short periods of time (41). Yet, all animals undergoing pilocarpine-induced SE develop spontaneous seizures and this is by far the animal model of epilepsy with the highest frequency of those seizures (Mello LE, unpublished results).

Another important SE model is the kainic acid model, which results from the intraperitoneal or intrahippocampal administration of KA, an agonist of the ionotropic glutamate receptor and a cyclic analog of L-glutamate. As it occurs after pilocarpine administration, the epileptiform discharges after KA administration originate in limbic structures and then propagate to other brain areas (38), mimicking human SE and promoting EEG changes similar to those seen in patients. As a disadvantage, KA and pilocarpine models result in high animal mortality and variable frequency and severity of spontaneous seizures among individual animals (34).

The maximal electroshock seizure, 6-Hz corneal psychomotor seizure, and pentylenetetrazol injection are other established models to induce acute seizures, but differently from what occurs in SE models, those seizures are self-limited and mostly used to test anti-seizure drugs, instead of PTE prevention.

## Models and physiopathological mechanisms

### Neurodegeneration

Although the mechanisms underlying the development of spontaneous recurrent seizures are not clearly understood, it is known that the degenerative process is one of the characteristics of brain pathologies related to acquired epilepsy (12).

The existence of relatively high numbers of animal models in which the developmental process and the morphological and molecular features of epilepsy in humans are reproduced has obvious advantages for research. Ideally, a model will efficiently reproduce the variety of tissue loss and damage detected in TBI. In human patients with severe TBI, besides local neuronal loss, subcortical neurodegeneration in the thalamus and hippocampus are also observed and may contribute to epileptogenesis (28).

Comparing two SE models, Covolan and Mello (42) observed prominent thalamic degeneration in the pilocarpine as well as in the KA model, although in the pilocarpine model, neuronal injury was more remarkable than that in the KA model in numerous areas in cortex, hippocampus, endopiriform nucleus, amygdaloid complex, and hypothalamus. On the other hand, there is no neuronal damage associated with the electrical kindling model (31). Furthermore, in the model of cortical injection of ferrous chloride, neurodegeneration is limited to the cortex (14). Moreover, the CCI model of TBI can cause different levels of injury severity and several degrees of cortical, subcortical, and hippocampal tissue damage that could lead to substantial neurodegeneration and tissue loss in the ipsilateral neocortex and adjacent hippocampus. The acute injury results in increased neuron loss, considerable lesion volumes, and more severe functional deficits (43) even for a long period (44).

As reviewed here, neurodegeneration is an important feature of almost all animal models considered in this section (Figure 1). There is no specific lesion profile or characteristic that is unique to any of the TBI models. More importantly, there are no neurodegenerative hallmarks that are only present in human PTE, even more so as the lesion event may affect a number of different cortical and subcortical regions.

### Neuroplasticity

As a consequence of brain injury, several neuroplastic changes might ensue. Neural plasticity involves both the proliferation and differentiation of newly-formed cells, in a process called neurogenesis, as well as alterations in the structure and function of neurons at different levels, from their morphology, subunit receptor composition, or neurotransmitter expression to synaptic connections. In this sense, neurogenesis (45) and axonal sprouting (46) have been extensively seen in chemically-induced SE models. The efforts to reestablish the neural connections could be beneficial, but could actually be adverse (or even an epiphenomenon) to the occurrence of spontaneous recurrent seizures in these animal models (46).

*Neurogenesis.* As previously mentioned, the latent period is a phase that occurs after the initial precipitating injury, lasting from days to weeks, in which the epileptogenic process occurs, leading to the development of later spontaneous seizures. Curiously, it was noticed that during the latent period a dramatic increase in cell proliferation in the subgranular zone of the dentate gyrus of the hippocampus and in subventricular zone of lateral ventricles occurs in several animal models of PTE (47). Jessberger and Parent (48) showed that epileptic activity induced by KA led to changes in the neuronal polarity, migration, and integration pattern of newly born granule cells, resulting in their ectopic location in the hilus. Zellinger et al. (49) observed that prolonged seizure activity induced by electrical stimulation resulted in a significant rise in the number of neuronal progenitor cells. Moreover, a significant increase in neurogenesis was observed in the granule cell layer of the dentate gyrus following pilocarpine-induced SE (45) and kindling stimulations (47).

Indeed, seizure activity is a neurogenic stimulus. Increased hippocampal neurogenesis is a common hallmark of most SE models, as well as TBI models (50) that develop epilepsy (Figure 1). Approximately 4-6 weeks after the initial insult, cell proliferation returns to normal levels (47,51).

Neuberger et al. (52) showed that the early increase in neurogenesis after FPI is transitory and is followed by a persistent and dramatic decrease in the number of newly generated cells. They suggest that early post-traumatic increases in neurogenesis negatively affect long-term events by diminishing the neurogenic potential of neural stem cells, which might in turn contribute to epileptogenesis. In addition, Hattiangady et al. (53) demonstrated that the intensity of neurogenesis declines in the chronic phase with the occurrence of spontaneous seizures.

It is believed that excessive neurogenesis may contribute to an aggravation of pathology, causing aberrant connectivity and enhanced excitability (54) and although seizures enhance neurogenesis, the survival of the newborn granular cells may decrease with increased seizure severity due to microglial activation (55).

While much work has been conducted regarding the neurogenic process in response to injury in animal models, only a few studies demonstrate these results in humans after TBI. In this sense, Zheng et al. (56) observed that neurogenesis occurs in the peri-damaged brain regions after TBI. However, they were unable to confirm whether the newly generated cells originated from local cortical progenitor cells or migrated from neurogenic regions after TBI. According to Richardson et al. (57), assessing the migration of neuroblasts from the subventricular zone to the damaged cortical area on TBI in the human brain becomes difficult due to the long distance between the two regions compared to the rostral migratory route of rodents. Chiaretti et al. (58) showed a significant up-regulation of doublecortin (DCX) level in the cerebrospinal fluid of children with severe TBI that may reflect an attempt at neuroprotection against the biochemical and molecular cascades triggered by traumatic insult.

There are no consistent reports on the effects of PTE in humans with regard to altered neurogenesis. Even more so, there is no consistent evidence indicating what would be the relevance of this altered neurogenesis after TBI in humans to the ensuing PTE.

*Mossy fiber sprouting.* Mossy fiber sprouting (MFS) was first described in patients with temporal lobe epilepsy and it became an important and frequent histopathological finding replicated in animal models. Mossy fibers normally extend to the hilus with projections to the excitatory and inhibitory interneurons and then run the stratum lucidum to synapse onto pyramidal neurons of the CA3 region. The projection of these axons under physiological conditions normally maintains a balance between inhibition and excitation, but in the epileptic brain, the MFS creates new recurrent excitatory circuits, projecting back to the molecular layer of the dentate gyrus (59). Normally, MFS is first detected within days after seizures or experimental lesions, develops by 2 weeks, and is long lasting. However, there has been controversy about the overall functional significance of sprouted excitatory circuitry as a contributing factor of spontaneous seizure development.

SE models, such as the pilocarpine and KA models, have been used to suggest an association between MFS and epileptogenesis (60). In contrast, Longo et al. (61) demonstrated that in pilocarpine-induced animals the prevention of MFS by cycloheximide (a protein synthesis inhibitor) does not interfere in the development of spontaneous seizures, despite preventing the MFS. In addition, Longo and Mello (62) also demonstrated that cycloheximide can block MFS induced by KA injection, without altering either the frequency or intensity of the behaviorally and/or electroencephalographically recorded ictal and interictal events.

Sutula et al. (63) demonstrated that even brief seizures of kindled rats induces long-lasting structural reorganization in neuronal circuits. MFS develops after a few brief seizures induced by kindling stimulation progressing with repeated seizures (64).

Although MFS is mostly observed in chronic SE models, it may also be seen in TBI models (Figure 1). According to Golarai et al. (65) weight-drop injury leads to a progressive bilateral MFS in the dentate gyrus. Substantial bilateral MFS could also be observed at both acute and chronic time points in FPI model, being more severe ipsilateral to FPI injury, presumably due to the more intense neuronal damage (25). Hunt et al. (66) also observed MFS ipsilateral to CCI injury at 8-12 weeks after insult. However, while some authors recognize that the MFS is qualitatively less abundant after TBI, compared with the robust sprouting observed after SE (67), according to Hunt et al. (23), MFS after CCI was similar to that observed in humans and other animal PTE models.

With the aim of evaluating the network excitability in the dentate gyrus after TBI, a series of studies using extracellular field recording have been done, but failed to regularly demonstrate epileptiform activity after TBI (23). In addition, Santhakumar et al. (68) showed that rats presented recovery a month after FPI, together with an early increase in granule cell layer extracellular excitability that was related to MFS. Therefore, Hunt et al. (66) suggest that the emergence of MFS may play a different functional role in the dentate gyrus after mechanical TBI compared to pharmacologically-induced PTE models.

In humans, MFS is frequently found in surgical epilepsy tissue (69). The mossy fibers terminals are distributed in the hippocampus of humans with epilepsy in a similar way as seen in the kindling model in rats (69). Swartz et al. (70) performed further histologic analyses in tissue from patients with a history of significant head trauma but without any other risk factor for epilepsy, from the California Comprehensive Epilepsy Program database. Ten of 11 hippocampal specimens presented some degree of MFS, and also of granule cell dispersion, both related with hilar neuron loss. Despite the observed reorganization of the mossy fibers, these authors could not correlate the presence of sprouting to the duration of epilepsy, and even less so whether it was a pre-existing condition to the head trauma.

In summary, despite the various studies that investigate MFS, its role in epileptogenesis remains controversial and unclear. MFS is a common finding in epilepsy but not all patients or animal models with spontaneous seizures develop MFS. Taking this into account, MFS has neither a pro-epileptogenic nor an anti-epileptogenic role.

### Neuroinflammation

The induction of cytokines after the occurrence of prolonged and acute seizures has been extensively studied in animal models of seizures and epilepsy. The activation of inflammatory pathways, which leads to the release of mediators, increases the severity of subsequent seizures and epileptogenesis, and supports the hypothesis that inflammation may play a lead role in the pathophysiology of seizures and the associated neuropathology in PTE (71).

Indeed, gliosis, microglia activation, and cytokine production, such as interleukin (IL)-1β and tumor necrosis factor (TNF)-α, and consequently neuroinflammation can change neuronal excitability by modulating receptor function and expression, perpetuating the chronic nature of epilepsy (71). Although neuroinflammation is considered to play a key role in the pathophysiology of epilepsy, what are (and whether there are) the critical neuroinflammatory processes underlying epileptogenesis is still unclear. Seizures induced either chemically or electrically could increase cytokines in rodent brain areas involved in the onset of epileptic activity (72). To that end, proinflammatory cytokines are induced in the brain also by kindled seizures (73). Also, Ambrogini et al. (74) demonstrated that KA-induced SE is capable of triggering neuroinflammatory processes in the hippocampus of rats characterized by astrogliosis and microglial activation, in addition to the expression of IL-1β and TNF-α.

Comparing the expression of IL-1β in microglia and astrocyte cells in two different SE models (electrical stimulation and pilocarpine-induced SE), Ravizza et al. (75) observed that during the acute phases of SE, the up-regulation of IL-1β was observed in both microglia and astrocytes, whereas only astrocytes showed enhanced immunostaining during epileptogenesis. In the chronic epileptic phase, IL-1β is still observed in astrocytes and also in microglia but only in the electrical stimulation model. These data indicate that the expression of IL-1β in astrocytes is continuous regardless of seizure frequency, whereas microglia expression depends on ongoing and severe epileptic activity, indicating that brain inflammation is a chronic process developing after the initial precipitating insult that can persist in epileptogenic tissue in the absence of ongoing seizure activity.

Moreover, increased expression of inflammatory genes (*IL-1β*, *IL-6*, *TNF-α*) in the hippocampus and amygdala has been reported in several PTE models such as KA (71) and lithium-pilocarpine (76), representing a generalized molecular response to seizures.

The neuroinflammation induced in TBI models has been reported as similar to that of SE models. According to Dalgard et al. (77), the expression of CXCL-1, IFN-γ, TNF-α, IL-4, and IL-13 are increased in ipsilateral cortex at 4 h after CCI. Increased expression of some cytokines were also reported 24 h after lateral FPI, both in the ipsilateral and contralateral cortex (78). Interestingly, the same research group reported similar magnitude of expression among the same cytokines 24 h after lateral FPI and after pilocarpine-induced SE (78,79). Specifically, both models showed the highest expression for CCL2 and similar expression levels for CCL3, CCL5, and TNF-α (78,79). In addition, Su et al. (80) using the FPI model reported that TBI may increase the expression of eotaxin, G-CSF, GM-CSF, GRO/KC, IFN-γ, IL-1α, IL-1β, IL-2, IL-4, IL-5, IL-6, IL-10, IL-12, p70, IL-13, IL-17, leptin, MCP-1, TNF-α, and VEGF in the area of cortical injury even 7 days after FPI. Residual level of neuroinflammation may also persist chronically (11,44,78).

It is well established that TBI in humans is associated with an inflammatory response that varies according to stimulus intensity, presence of blood in the brain parenchyma, and the presence of penetrating injury, among other aspects (81). However, causal links between inflammatory signaling cascades taking place after TBI and the emergence of seizures and the development of epilepsy have yet to be more firmly established.

In summary, it is well-established that seizures promote an inflammatory immune cascade, although emerging evidence also suggests that neuroinflammation may also be causal to seizures and epilepsy by inducing changes in neuronal function and connectivity, leading to regional hyperexcitability and subsequent seizure susceptibility. Nevertheless there is no distinctive feature that characterizes these processes among PTE models, which in general, display similar cascades of events (Figure 1).

## Antiepileptic drugs (AEDs)

It is known that people who suffer TBI have increased acute seizure susceptibility. In this sense, a series of AEDs are routinely administered in the clinic, soon after TBI, to control acute seizures (occurring within 1-2 weeks from injury). In addition, there are also many clinical trials using known AEDs, like carbamazepine, valproic acid, phenobarbital, phenytoin, and other compounds aimed at preventing the development of PTE, but all have failed to prove efficacy (28). Therefore, the use of experimental models to verify the effectiveness of already known AEDs and test new compounds on PTE prevention is of extreme importance.

The kindling model has long been used to study the effects of anticonvulsant drugs on seizure development. Diazepam is more effective at blocking amygdala-kindled seizures compared to seizures induced by cortical stimulation, which are better blocked by procaine hydrochloride and diphenylhydantoin. Phenobarbital is effective in the suppression of hippocampal and amygdala seizures (30). To make the topic even more complex, a single exposure to carbamazepine or lamotrigine 48 h after stimulation leads to a decrease in the capacity of these AED to mitigate further seizures evoked after discharges (34). According to Löscher (82), in the amygdala-kindling, low doses of lamotrigine during the development phase leads to a reduced anticonvulsant response to the drug in fully kindled rats. Lamotrigine-refractory kindled rats are resistant to topiramate, carbamazepine, and phenytoin, but not felbamate, valproate, and retigabine. During the kindling acquisition phase, some antiepileptogenic drugs (i.e., valproate) given before each stimulus could be sufficient to mitigate or retard the kindling. Song et al. (33) compared the effects of three AEDs in mice submitted to kindling models. Levetiracetam and phenytoin abolished spontaneous recurrent seizures in 6 of 6 and 5 of 6 mice, respectively. Lorazepam, in turn, was effective in decreasing the motor seizures severity in all mice, but it was not enough to reduce the duration and incidence of associated afterdischarges in the hippocampus.

Also, many compounds have been tested in TBI models. According to Pitkanen and McIntosh (28), AEDs used in clinics, such as carbamazepine, phenobarbital, valproate, phenytoin, clonazepam, zonisamide, primidone, and ethosuximide, can all suppress spontaneous seizures induced by ferric chloride, but again, with no translational benefit proven.

In the FPI model, rats acutely treated with tacrolimus showed a reduction in the number of non-convulsive seizures compared to untreated rats, even presenting a similar degree of cortical atrophy (6). In addition, slices from rats treated with felbamate after FPI exhibited long-term potentiation in CA1, which was suppressed in untreated animals. This fact suggests a possible neuroprotective property of felbamate against TBI (25). The administration of topiramate 30 min after FPI proved beneficial to sensorimotor behavior, even though it was not favorable on learning or neuronal survival. Other drugs used after FPI such as lacosamide had no effect on lesion size, anatomical damage, motor impairment, or functional recovery (83). According to Dash et al. (84), the administration of valproate starting at 30 min or 3 h after CCI promoted improvement of blood-brain barrier (BBB) integrity, decrease of hippocampal dendritic damage, improved spatial memory and motor function, and lessened cortical contusion volume. High doses of ethosuximide and phenytoin administered 30 min after penetrating ballistic-like brain injury attenuated spontaneous non-convulsive seizures, but lower doses failed to have the same effect (85). The clinical relevance of such findings, however, is hampered by the extremely short time interval between injury and drug administration.

Leite and Cavalheiro (86) tested the effect of five AEDs in the pilocarpine model. Phenobarbital, phenytoin, and carbamazepine were effective against spontaneous seizures. Valproic acid was also effective against spontaneous seizures, but only at the high doses. Last, but not the least, ethosuximide was not effective against spontaneous seizures. In accordance, Glien et al. (87) also showed that levetiracetam decreases spontaneous recurrent seizures frequency in the rat pilocarpine model.

In the KA model, valproate, carbamazepine, and lamotrigine were effective to dose-dependently suppress hippocampal paroxysmal discharges (HPDs) and modified EEG activity and behavior. At high doses, levetiracetam and pregabalin were effective to suppress HPDs, but did not change any behavior or EEG activity. Furthermore, dose-dependent diazepam, tiagabine, phenobarbital, and vigabatrin suppressed HPDs, but did no other change (88).

It is now clear that the so-called AEDs are in fact drugs that affect the expression of seizures but do not affect the development of epileptogenesis. Despite their name, AEDs do not prevent epilepsy but only its expression (e.g., seizures). Indeed, as discussed in the previous sections of this review, the lesion that may trigger PTE, regardless of its nature, sets in motion a similar cascade of events that overlaps with other models of epilepsy. Thus, drug treatment tested on these other epilepsy models with a higher frequency of recurrent spontaneous seizures would ultimately have great predictive value for PTE (even when not using isomorphic models to TBI).

To date, neither TBI or SE models (but see below) have provided evidence of compounds that would effectively alter epileptogenesis after TBI. The predictive capability that could be expected from models with a stronger etiological basis has not yet been demonstrated. The fact that the emergence of spontaneous seizures is a rare event in TBI models does not help in providing a platform for testing of such compounds.

Research from our group has provided evidence that anticholinergic agents might provide an effective means to modify disease progression in the pilocarpine model of epilepsy (89,90). The administration of scopolamine starting 3 h after the onset of pilocarpine-induced SE was effective in altering epileptogenesis (90). Similarly, in a more extensive study, the administration of biperiden (another cholinergic antagonist) was also shown to be effective in altering disease progression in the same model (89). We concluded that anti-muscarinic agents or, more broadly, drugs that interfere with plastic processes may be potentially relevant in the quest for disease-modifying agents that may diminish the burden of PTE.

## Final remarks

We put forward the suggestion that the ideal model to investigate potential therapies against PTE has yet to be defined. In fact, for numerous aspects, TBI models mimicking the mechanical traumatic injury such as CCI, FPI, blast, and so on are the most faithful to recapitulate the sequence of events after TBI. However, the diminished capacity of those models to easily promote PTE also hampers their ability to provide more useful instruments in the screening of new compounds that may influence epileptogenesis. Here, we addressed the main differences and similarities between typical TBI models, kindling, and SE models with regard to the emergence of spontaneous seizures, seizure response to drugs, neuroinflammation, and other features often studied in animal models and human epilepsy, most notably PTE. Currently, the main practical application of any model of PTE is that of providing a means to prevent or modify disease progression after TBI. Here, we provide evidence that, except for the causative (etiological) aspect of TBI models, there is no distinctive pathological feature that is exclusively present in those models and not in the kindling or SE models. We put forward the hypothesis that the use of various modeling strategies, and not only those restricted to mechanical traumatic injury, might allow for a broader understanding of the different mechanisms of seizure induction, propagation, and occurrence and could help the search for biomarkers and new drugs to prevent PTE.
